# Metal-Binding Proteins Cross-Linking with Endoplasmic Reticulum Stress in Cardiovascular Diseases

**DOI:** 10.3390/jcdd10040171

**Published:** 2023-04-17

**Authors:** Kejuan Li, Yongnan Li, Hong Ding, Jianshu Chen, Xiaowei Zhang

**Affiliations:** 1 Department of Cardiology, Lanzhou University Second Hospital, Lanzhou University, Lanzhou 730031, China; 2 Department of Cardiac Surgery, Lanzhou University Second Hospital, Lanzhou University, Lanzhou 730031, China; lyngyq2006@foxmail.com

**Keywords:** Ca^2+^, cardiovascular disease, endoplasmic reticulum stress, metal-binding protein, UPR

## Abstract

The endoplasmic reticulum (ER), an essential organelle in eukaryotic cells, is widely distributed in myocardial cells. The ER is where secreted protein synthesis, folding, post-translational modification, and transport are all carried out. It is also where calcium homeostasis, lipid synthesis, and other processes that are crucial for normal biological cell functioning are regulated. We are concerned that ER stress (ERS) is widespread in various damaged cells. To protect cells’ function, ERS reduces the accumulation of misfolded proteins by activating the unfolded protein response (UPR) pathway in response to numerous stimulating factors, such as ischemia or hypoxia, metabolic disorders, and inflammation. If these stimulatory factors are not eliminated for a long time, resulting in the persistence of the UPR, it will aggravate cell damage through a series of mechanisms. In the cardiovascular system, it will cause related cardiovascular diseases and seriously endanger human health. Furthermore, there has been a growing number of studies on the antioxidative stress role of metal-binding proteins. We observed that a variety of metal-binding proteins can inhibit ERS and, hence, mitigate myocardial damage.

## 1. Introduction

Cardiovascular disease is a non-communicable disease that poses a serious threat to human health and affects one’s quality of life. It is the main cause of morbidity and mortality worldwide [1,2]. Despite substantial improvements in cardiovascular disease-related mortality due to improved global quality of life and medical technology, population growth and aging have outpaced these advancements worldwide [3]. These trends have led to a 40% increase in the total number of cardiovascular disease-related deaths since 1990 [4]. Therefore, it is crucial to investigate the mechanism of cardiovascular disease and effective treatment measures. The heart is a high-energy organ capable of metabolizing different substrates, including fatty acids (FAs), glucose, lactate, and ketones [5].

The endoplasmic reticulum (ER) is the organelle that synthesizes and processes approximately one-third of the eukaryotic proteome, including secreted proteins, plasma membrane proteins, and proteins transported to other inner membrane systems [6]. In addition, the ER is involved in calcium signaling, lipid and steroid synthesis, the metabolism of drugs and xenobiotics, the regulation of gene expression and energy metabolism, and the transmission of signals to the nucleus, cytoplasm, mitochondria, and plasma membrane [7,8,9,10]. Thus, oxidative stress, reactive oxygen species (ROS) production, viral infection, pH reduction, drugs, inflammatory responses, and metabolic disorders disrupt ER homeostasis, leading to ER stress (ERS), which in turn causes oxidative damage and validation responses, leading to cardiovascular disease, cancer, metabolic diseases, and neurodegenerative diseases [11]. In turn, pathophysiological factors associated with cardiovascular diseases, such as metabolic disorders, hypoxia, and inflammation, can also cause ERS and aggravate cardiovascular injury, producing a vicious circle.

Trace elements are essential for maintaining health and performing normal life activities. Since the majority of these trace elements function as metal-binding proteins, interest in the function of these proteins in the body has also increased. When metal ion homeostasis is disturbed, the body may experience oxidative stress and an increased formation of ROS, exceeding the body’s antioxidant defense, as well as other impacts that can cause DNA damage, lipid peroxidation, protein modification, and other effects, ultimately causing many diseases [12]. Numerous studies have proven that metal-binding proteins can inhibit ROS production, although there are limited studies on the relationship between metal-binding proteins, ERS, and cardiovascular diseases. We hypothesized that metal-binding proteins can inhibit ROS production, thereby inhibiting oxidative stress and ERS and playing an organ-protective role. For example, metallothionein (MT) acts as an antioxidant by binding ROS to its sulfhydryl groups, thereby playing a protective role in disease models of various organs [13,14]. Zinc is an important component of many proteins involved in combating oxidative stress. Studies have demonstrated that a zinc deficiency may exacerbate DNA damage by damaging DNA repair mechanisms [12,15,16].

In this context, we summarize the factors that induce ERS activation in cardiovascular diseases and their related consequences, as well as the inhibitory effects of some antioxidants, such as plant extracts, metalloproteins, and other antioxidant enzyme systems, on ERS. We further discuss potential genetic targets and new drugs that could be developed by interfering with the ERS pathway to alleviate the occurrence and development of cardiovascular diseases.

## 2. ERS and UPR

The proteostasis network in the ER ensures that misfolded or conformationally unstable proteins are targeted for degradation, thereby preventing their transport through the secretory pathway. Failure of the ER quality control system may lead to the accumulation of misfolded proteins in the ER or the release of toxic, aggregable proteins. The accumulation of unfolded or misfolded proteins in the ER induces the unfolded protein response (UPR) [17]. The UPR is a complex stress response pathway that activates factors that reconstitute protein homeostasis, leading to cell adaptation or apoptosis to regulate ER homeostasis. UPR is an important determinant of cellular activity, as it is involved in autophagy, apoptosis, metabolic changes, inflammation, tumor immune infiltration, invasion and metastasis, and angiogenesis [18].

**Figure 1:**
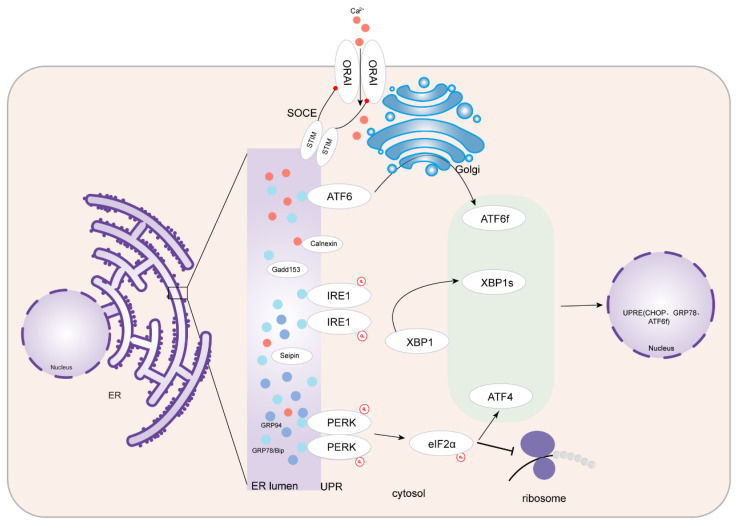
Endoplasmic reticulum stress. UPR’s three branches including PERK-eIF2a-ATF4, IRE1-XBP1, and ATF6 in ERS, and the process of SOCE regulates calcium homeostasis in ER. ERS, endoplasmic reticulum stress; UPR, unfolded protein response; PERK, protein kinase RNA-like ER kinase; eIF2a, eukaryotic translation initiation factor 2a; ATF4, activating transcription factor 4; IRE1, inositol-requiring protein 1; XBP1, spliced X-Box binding protein 1; ATF6, activating transcription factor 6; ORAI, calcium release-activated calcium channel protein I; STIM, stromal interaction molecule; SOCE, store-operated calcium entry; CHOP, C/EBP homologous protein; UPRE: unfolded protein response element; GRP78, glucose regulating protein 78; GRP94: glucose regulating protein 94.

The UPR is known to have three branches, including protein kinase RNA-like ER kinase (PERK)-eukaryotic initiation factor 2α (eIF2a)-activating transcription factor 4 (ATF4), inositol-requiring enzyme type 1-X-box-binding protein 1 (IRE1-XBP1), and ATF6 [19,20]. (1) The PERK-eIF2a-ATF4 pathway: PERK is a kinase that oligomerizes and phosphorylates itself after activation. p-PERK acts downstream to phosphorylate eIF2α, and p-eIF2α inhibits ribosome function, causing most protein translation to stop and reduce protein synthesis to restore cell homeostasis. It causes cell growth cycle arrest. However, the translation of ATF4, a transcription factor that can increase the expression of numerous cytoprotective genes, is not inhibited. ATF4 translocates into the nucleus, where it binds to UPR-related proteins (such as the C/EBP homologous protein, CHOP) to trigger apoptosis. (2) The IRE1-XBP1 pathway: Similar to PERK, when IRE1 is activated, p-IRE1 induces the splicing of the mRNA encoding the transcription factor XBP1 to form the actual transcriptor XBP1s. XBP1s translocates into the nucleus bound to the promoter of the UPR gene, causing downstream gene expression. (3) The ATF6 pathway: Following the separation of ATF6 and 78 kDa glucose-regulated protein (GRP78), the Golgi localization sequences of ATF6 become exposed. After activation, ATF6 enters the Golgi, where it is cleaved to form ATF6f. ATF6f is then translocated to the nucleus, where it binds to the promoter of the UPR gene to induce the expression of GRP78, CHOP, XBP1, and other genes [21,22,23,24]. These cascades are maintained at rest by the ER protein’s significant other, GRP78, which interacts with the luminal domains of three ER transmembrane molecules (namely PERK, IRE1, and ATF6) to stabilize them on the ER membrane’s surface. An abundant accumulation of misfolded or unfolded proteins in large quantities in the ER cavity activates ERS when protein dysfunction occurs. During ERS, BiP/GRP78 exhibits a higher affinity for these proteins and preferentially binds to the hydrophobic region on the misfolded proteins in the ER, thereby assisting these proteins to fold correctly. Simultaneously, GRP78 is separated from ER transmembrane proteins, activating ER transmembrane proteins and downstream signals. By inhibiting protein translation or through the ubiquitin protease system degradation pathway, it increases ER-related protein degradation and maintains the normal functioning of the ER. However, if this process fails, ERS persists, or UPR is unregulated, it triggers the increased expression of the proapoptotic marker CHOP, leading to cell cycle arrest and the induction of apoptosis and death [25,26] (Figure 1).

## 3. ERS and Metal-Binding Proteins

Trace metal elements are essential for the normal biological functioning of the body, although they do not account for a large proportion of the human body. Trace metal elements mainly play a role by binding to proteins. Metal-binding proteins are the first level of antioxidant defense, of which free iron and copper are the most important catalysts for the formation of free radicals. To combat the oxidative stress caused by ROS, organisms have evolved a variety of enzymatic antioxidants, mainly including superoxide dismutase (SOD), catalase (CAT), and glutathione peroxidase (GSH-Px) [27,28,29]. Most trace metal elements combine with proteins and play an important role in the occurrence and development of cardiovascular diseases. In recent years, research on the relationship between the two has attracted great interest (Table 1).

### 3.1. Selenium Protein

Selenium is a bioessential trace element that is incorporated into the selenium protein as selenocysteine and plays an important role in many biological processes. There are seven selenomins in the ER, including selenomin 15 (Sep15), deiodinase 2, selenomin S (SelS), selenomin N (SelN), selenomin K (SelK), selenomin M, and selenomin T (SelT) [55]. The ER-resident selenoprotein is an antioxidant involved in inflammation, oxidative stress, ERS, ER-associated protein degradation (ERAD), and the regulation of intracellular calcium homeostasis [56,57]. The accumulation or absence of selenium impairs its normal function. In myocardial tissue, selenium and selenoproteins are potential determinants of pathological cell hypertrophy, which is characterized by enlarged cardiomyocytes, increased protein synthesis, increased glucose dependence, and increased myocardial contractility. Several studies have revealed that miR-200a-5p inhibits the expression of selenoprotein P (Sepp1), SelN, SelT, and Sep15 and increases the expression of thioredoxin reductase 2 (Txnrd2) and Txnrd3, causing glucose transport dysfunction and glucose level increases, and ultimately leading to myocardial cell hypertrophy [58].

SelS is a selenium-sensitive selenium protein with an anti-ERS function. Reducing SelS expression will not cause ERS, but it will reduce cells’ ability to resist ERS. When there are excess unfolded or misfolded proteins during ERS, cells immediately increase SelS expression and accelerate the ERAD process. SelS forms a complex with cytoplasmic valine-containing proteins, derlins, SelK, and E3 ligases to transport unfolded or misfolded proteins from the ER to the cytoplasm. These proteins are degraded by the ubiquitin protease system to relieve ERS [59]. SelK can also promote ERAD and reduce the accumulation of misfolded proteins and the intensity of UPR, thus becoming a negative regulator of ERS signaling. Silencing the SelK gene will exacerbate ERS-mediated UPR, ROS production, apoptosis, and autophagy [60]. However, SelK has also been reported to promote the development of atherosclerosis. Moreover, SelK acts as a cofactor of DHHC6 (the letters represent the amino acids aspartic acid, histidine, histidine, and cysteine in the catalytic domain) in the ER to regulate the palmitoylation of triphosphate receptor and CD36 in immune cells and macrophages, respectively [61]. Increased SelK gene expression promotes the subcellular transport of fatty acid transferase (CD36) because CD36 mediates the uptake of long-chain fatty acids in myocardial tissue, so SelK overexpression exacerbates lipid accumulation in cells and promotes foam cell formation and atherosclerosis.

**Figure 2:**
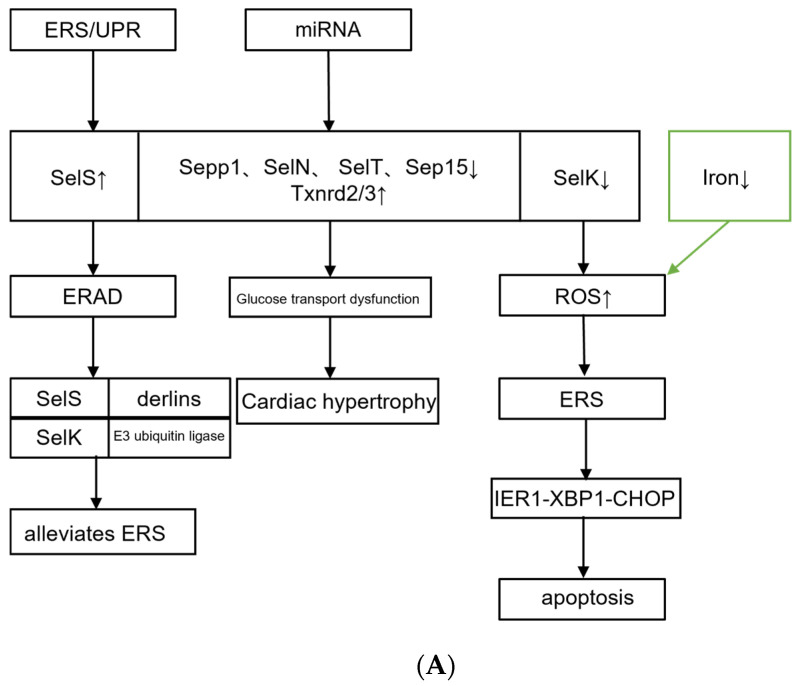

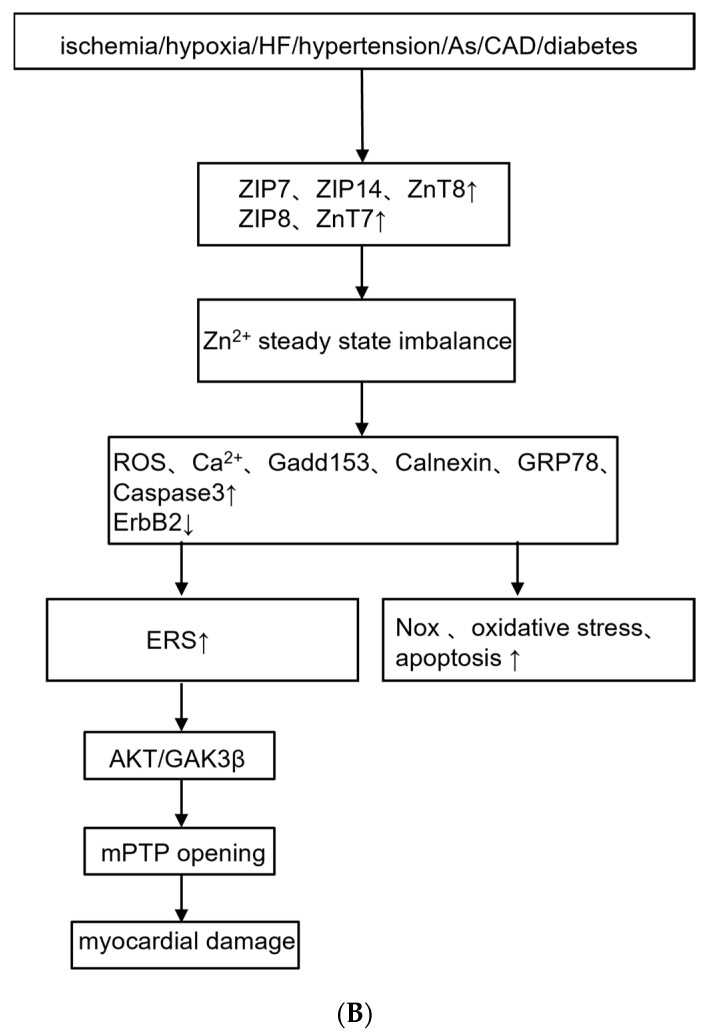
(**A**) **Selenium—related binding proteins and endoplasmic reticulum stress.** (**B**) **Zinc-related binding proteins and endoplasmic reticulum stress.** The absence or increase in trace metals in the body can cause adverse effects through ERS, and metal-binding proteins inhibit oxidative stress. Sels, selenoprotein; Sepp1, recombinant selenoprotein P1, plasma; Sep15, 15 KDa selenoprotein; ERAD, ER-associated degradation; IRE1, inositol-requiring protein 1; XBP1, spliced X-Box binding protein 1; CHOP, C/EBP homologous protein; ZnTs, zinc transporters; ZIPs, zinc–iron transporters; GRP78, glucose regulating protein 78; Nox, NADPH oxidase; ErbB2, Erb-B2 receptor tyrosine kinase 2. The up arrows indicate increase and the down arrows indicate decrease.

In an experiment in which vitamin E and selenium were added to the diets of broilers, the results showed a significant increase in the serum iron and serum copper of the broilers. Furthermore, the addition of vitamin E or vitamin E plus selenium increased the activity of antioxidant-related enzymes and reduced malondialdehyde (MDA), which represented lipid peroxidation in broiler liver tissue. The most well-known enzymatic antioxidants are SOD, CAT, and GSH-Px. They are the first-line defense against ROS. With the increase in dietary selenium levels, the expression levels of the antioxidant enzymes CAT, GSH-Px, and SOD in broiler liver tissue increase. In this experiment, it was demonstrated that vitamin E and/or selenium can not only be used as exogenous antioxidants to prevent oxidative damage by scavenging free radicals and superoxide disproportionation but also as gene modulators to regulate the expression of endogenous antioxidant enzymes [27]. The subject of this experiment was broiler chickens, and the degree of conservation of their genetic sequences is somewhat different from that of mammals. However, we suspect that selenoproteins also have the same trend in mammals. Therefore, further experimental verification is required to clarify their mechanism of action. Increased ROS production in cardiomyocytes mediates endoplasmic reticulum stress and causes cardiomyocyte injury [62,63]. ROS is inhibited by vitamin E and selenium to improve antioxidant-related enzyme activities. Therefore, we speculate that vitamin E and selenium also have certain effects on ERS, but there are few reports in the relevant literature (Figure 2).

### 3.2. Zinc Transporters

The trace element zinc in the human body is necessary for normal cell structure and function. Normal cell homeostasis requires appropriate zinc transport and storage. Zinc lacks redox capability; however, it serves as a cofactor for numerous enzymes and proteins that have antioxidant, anti-inflammatory, and anti-apoptotic properties. It functions through specific zinc transporters [46]. Myocardial stimulators such as ischemia and infarction can cause proteins to release zinc and cause further myocardial damage [46]. It has been demonstrated that zinc levels are usually significantly reduced in conditions such as atherosclerosis, heart failure, coronary artery disease, angina pectoris, and cardiac ischemia [64,65]. The imbalance of Zn^2+^ homeostasis caused by alterations in Zn^2+^ transporters may be related to the induction of ERS and apoptosis in hypertrophied hearts. In the myocardial tissue of rats in the transverse aortic constriction group, the expression levels of ERS markers GRP78, CHOP/Gadd153, and calnexin were significantly higher, and the expression of Zn^2+^ transporters ZIP7, ZIP14, and ZnT8 was significantly increased, while the levels of ZIP8 and ZnT7 were decreased [46]. The concentration of Zn^2+^ and the expression levels of ZIP14 and ZnT8 in the left ventricular tissue of patients with heart failure increased significantly, while the level of ZIP8 decreased [65]. Therefore, the role of zinc homeostasis in the pathophysiology of cardiovascular disease is critical.

One of the mechanisms of the toxic effects of Zn^2+^ homeostasis imbalance on the human diabetic heart, dilated cardiomyopathy, myocardial ischemia, and heart failure is related to ERS [65,66,67]. The dysregulation of cellular Zn^2+^ disrupts Zn^2+^ homeostasis in vivo, causing dysfunctional mitochondria and ERS, the disruption of normal ER/mitochondrial crosstalk and mitosis, elevated ROS, and metabolic dysfunction [68]. Exogenous zinc supplementation has been reported to reduce myocardial infarction size, inhibit ERS and autophagy processes, and thereby protect the heart [66,68]. Hypoxia/reoxygenation reduce intracellular Zn^2+^ concentration, activate caspase-3, and increase tyrosine kinase receptor erythroblastic leukemia viral oncogene homolog 2 (ErbB2) degradation, which is involved in the regulation of embryonic development, cell proliferation, apoptosis, and other physiological and pathological processes, and ERS in H9C2 cells. Moreover, zinc depletion with Zn^2+^ chelators *N*,*N*,*N*′,*N*′-tetrakis (2-pyridylmethyl)-ethylenediamine (TPEN) resulted in decreased ErbB2 and increased apoptosis, the upregulation of NADPH oxidase 2 (Nox2) mRNA, and increased oxidative stress and ERS. Zinc supplementation inhibited Nox2 mRNA, reduced oxidative stress and ERS, and reduced cell death [48]. The myocardium of high-fat diet and streptozotocin-induced diabetic mice contained significantly more autophagosome marker light chain 3 and GRP78 proteins. All these alterations were significantly reduced with zinc supplementation [49]. Zinc supplementation during reperfusion inhibits oxidative stress, thereby protecting cardiomyocyte mitochondria [69]. In hepatocytes, it was observed that excessive copper can lead to the accumulation of unfolded or misfolded proteins in the lumen of the ER, inducing ERS and oxidative stress. Exogenous use of zinc acetate can inhibit ERS and hepatotoxicity, including DNA damage and apoptosis [70].

An important target for myocardial protection is the mitochondrial permeability transition pore (mPTP). Zn^2+^ reduces ERS by gating and regulating mPTP, which plays an important role in myocardial protection. Studies have reported that tunicamycin (TM) treatment can activate ERS and significantly increase the level of Ca^2+^ and mitochondrial ROS in H9C2 cardiomyocytes. Zn^2+^ significantly reduces the protein level of the ERS marker protein GRP78/94 and inhibits the increase in intracellular Ca^2+^ and ROS [71]. ERS is activated during myocardial I/R. Administration of the ERS inhibitor taurodeoxycholic acid significantly decreased GRP78 expression early after the onset of reperfusion, which was reversed by TPEN. Simultaneously, tauroursodeoxycholic acid reduced myocardial infarct size, which was reversed by the mPTP opener atractyloside. In addition, ERS may regulate mPTP opening through the AKT/glycogen synthase kinase-3β (GSK-3β) signaling pathway, thereby impairing cardiac contraction [50]. Zn^2+^ protects the myocardium by closing mPTP and inhibiting ERS. Therefore, a well-controlled Zn^2+^ concentration can be used as an important therapeutic method for the prevention or treatment of cardiovascular diseases. Resveratrol, a natural polyphenol substance, mobilizes intracellular Zn^2+^ concentration and prevents mPTP opening via the extracellular signal-regulated kinase (ERK)/GSK-3β signaling pathway, thereby protecting heart cells from the effects of ERS [72].

In addition, there are other metal-binding proteins, such as manganese (Mn), which are also essential trace elements in the human body. They exist in various cells and tissues and are crucial in scavenging free radicals and inhibiting oxidative stress. It has been demonstrated that Mn enhances the activity of MnSOD, which is a major antioxidant enzyme [73]. During chick embryo development, Mn plays an anti-apoptotic role by inhibiting ROS generation and the ERS pathway and increasing MnSOD enzyme activity to alleviate heat stress-induced apoptosis, thereby protecting cardiomyocytes from oxidative stress [74]. Furthermore, SOD overproduction is associated with atrial electrical and structural remodeling and elevated levels of MnSOD in circulating plasma have been reported to be an independent risk factor for paroxysmal atrial fibrillation [28]. Iron plays an important role in erythropoiesis and oxygen transport [75,76,77]. Ferritin is a requirement for numerous biological activities, such as oxygen transport, mitochondrial respiration, the metabolism of intermediates and derivatives, nucleic acid replication and repair, cellular defense, and cellular signaling pathways [78]. Iron deficiency (ID) activates the ERS response by inducing mitochondrial oxidative stress. The IRE1-XBP1-CHOP signaling pathway is activated, which accelerates the apoptosis of arterial smooth muscle cells in the aortic middle layer, thereby promoting the formation of aortic middle layer degeneration [78]. In addition, ID can cause chronic heart failure and other cardiovascular complications [79,80,81,82]. Ceruloplasmin and MT, discussed below, are antioxidants that protect the myocardium by inhibiting oxidative stress.

## 4. Metal-Binding Proteins Cross-Linking with ERS in Cardiovascular Diseases

ER homeostasis is closely related to normal cardiovascular function. Various pathological stimuli induce ERS, resulting in the emergence of cardiovascular diseases such as atherosclerosis, ischemic heart disease, hypertension, heart failure, and congenital heart disease. Cardiovascular function is protected by early UPR and ERS; however, prolonged or excessive ERS or UPR can cause cytotoxicity and apoptosis. In recent years, ERS has become an important mechanism in the study of the pathogenesis of cardiovascular diseases. An effective approach for the treatment of heart diseases is cardiac therapy based on ERS regulation.

### 4.1. Atherosclerosis

Endothelial cells (ECs) can release proinflammatory mediators and recruit monocytes to accumulate in the vascular wall, which is one of the major factors in the development of atherosclerosis [83]. Atherosclerosis is a chronic inflammatory disease characterized by the infiltration of activated monocytes or macrophages and T cells in the intima and by the production of numerous proinflammatory factors by cells within the lesion [84]. In addition, the lesion exhibits an increased expression of adhesion molecules, such as intercellular adhesion molecule (ICAM)-1 and vascular cell adhesion molecule (VCAM)-1, on the surface of ECs, which is essential for leukocyte migration to the subendothelium of atherosclerotic lesions [84]. ERS and the UPR contribute to atherosclerotic plaque formation [85].

**Figure 3:**
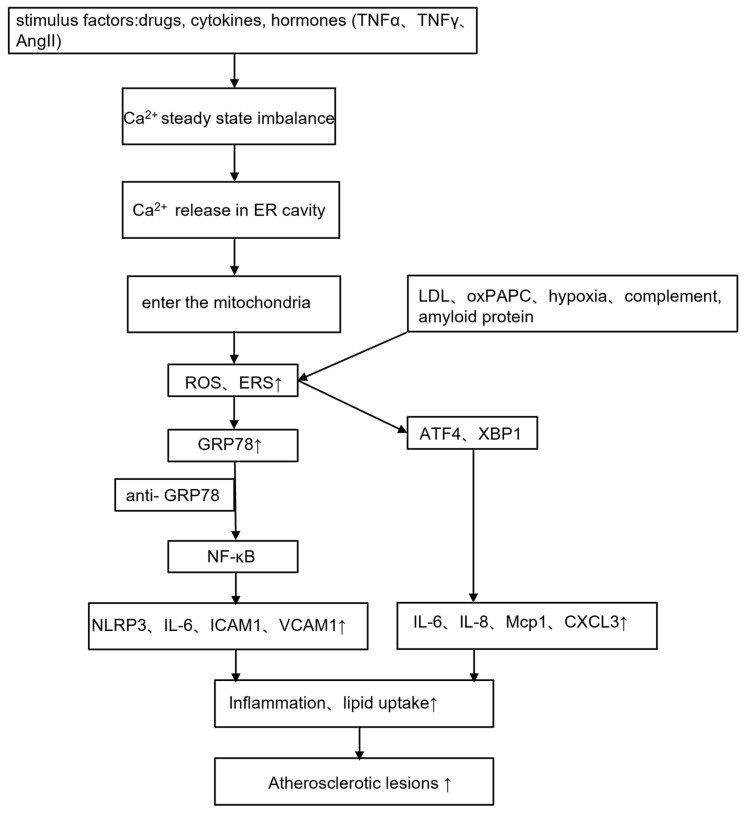
Endoplasmic reticulum stress and atherosclerosis. Atherosclerosis is an inflammatory disease. Stimulatory factors in the body cause an imbalance in calcium homeostasis, which subsequently causes ERS, activates NF-κB, and mediates the inflammatory response. In addition, ERS at the lesion causes macrophages to phagocytose a large number of lipids, aggravating atherosclerotic lesions. TNFα, tumor necrosis factor α; INF-γ, interferon-γ; AngII, angiotensin II; LDL, low-density lipoprotein; oxPAPC, oxidized 1-palmitoyl-2-arachidonyl-sn-3-glycero-phosphorylcholine; NF-κB, nuclear factor kappa-B; NLRP3, NLR family pyrin domain containing 3; IL, interleukin; ICAM1, intercellular adhesion molecule 1; VCAM1, vascular cell adhesion molecule 1; MCP-1, monocyte chemoattractant protein-1; CXCL3, C-X-C Motif Chemokine Ligand 3; GRP78, glucose regulating protein 78; XBP1, spliced X-Box binding protein 1; ATF4, activating transcription factor 4. The up arrows indicate increase and the down arrows represent decrease.

The ER functions as a checkpoint for intracellular Ca^2+^ storage and preserves most of the biosynthetic enzymes necessary for cell lipid synthesis. Some drugs, cytokines, and hormones, such as tumor necrosis factor α (TNFα), interferon-γ (IFN-γ), angiotensin II, and lipids, can affect calcium homeostasis in the ER and can cause calcium release in the ER lumen. Ca^2+^ enters the mitochondria, activates mitochondrial metabolism, and causes excess production of ROS and ERS. The disruption of Ca^2+^ homeostasis caused by cytosolic Ca^2+^ overload and the depletion of Ca^2+^ in the ER lumen may be factors causing ERS and misfolded protein synthesis, thereby activating the inflammatory response, the antioxidative stress response, apoptosis, and other stress response pathways [86] (Figure 3).

Ca^2+^ storage in the ER is detected by stromal interaction molecule 1 (STIM1) and STIM2 sensors on the ER membrane [87]. Cardiomyocytes monitor Ca^2+^ homeostasis through store-operated calcium entry (SOCE), a process that causes extracellular calcium influx and STIM. When the Ca^2+^ stored in the ER is depleted, Ca^2+^ is released from the Ca^2+^ binding domain of STIM. This triggers STIM dimerization and movement to the position closest to the ER and plasma membrane, where it interacts with the calcium release-activated calcium channel protein (ORAI), activates SOCE, and triggers a Ca^2+^ influx mechanism that allows Ca^2+^ to enter the ER across the membrane, thereby regulating Ca^2+^ homeostasis in the ER [88]. An imbalance in ER–Ca^2+^ homeostasis causes ERS, resulting in the over-secretion of GRP78 [89]. GRP78 is antigenic and causes the body to produce anti-GRP78 autoantibodies. Simultaneously, ERS induces GRP78 translocation to the cell surface [90,91], which binds to anti-GRP78 antibodies to activate nuclear factor kappa-B (NF-κB). NF-κB binds to the ICAM-1 and VCAM-1 promoters on the surface of ECs, then induces the transcription of NF-κB target genes (interleukin (IL)-6, ICAM-1, VCAM-1, and so on) [92,93,94], which promotes the occurrence of the inflammatory response and aggravates atherosclerotic lesions.

Lipid metabolism disorder is another important cause of atherosclerosis, especially the increased level of low-density lipoprotein (LDL). In atherosclerotic lesions in ApoE-/- mice, it was observed that minimally oxidized LDL and its main bioactive component, oxidized 1-palmitoyl-2-arachidonoyl-sn-3-glycero-phosphorylcholine, can cause ERS and misfolded or unfolded protein accumulation in the ER, resulting in human aortic EC activation of the UPR pathway. Furthermore, the transcription factors ATF4 and XBP1 in the UPR mediate the expression of inflammatory mediators IL-8, MCP1, IL-6, and CXCL3 and regulate the folding, maturation, and degradation of ER proteins [95]. Macrophages engulfing large amounts of cholesterol can also activate the UPR and the pro-apoptosis marker CHOP, resulting in increased apoptosis.

The nucleotide-binding domain (NOD)-like receptor protein 3 (NLRP3) inflammatory corpuscle is believed to be the link between lipid metabolism and inflammation in atherosclerosis. The occurrence of atherosclerosis is thought to be caused by almost all the factors that initiate the NLRP3 inflammatory corpuscle (including ROS, ox-LDL, hypoxia, complement, amyloid, and misfolded proteins) [83]. In addition, ERS can activate the NLRP3 through increased ROS, ER-Ca^2+^ release, and NF-κB activation, thereby triggering the inflammatory response [18].

### 4.2. Heart Failure

The stimulator of IFN genes (STING) plays an important role in the collective immune process. ERS-related proteins such as PERK were detected in the hearts of mice with myocardial hypertrophy caused by aortic banding. Furthermore, expression levels of PERK, eIF2α, and IRE-1α proteins were suppressed in mice with the STING gene knocked out. This demonstrates that ERS is involved in the process of cardiac hypertrophy caused by cardiac overload [96]. Studies have reported that the inhibition or deletion of silent information regulator 1 (SIRT1) can induce cardiomyocyte apoptosis and enhance cardiac dysfunction through the ERS pathway. Conversely, the SIRT1-activating compound STAC-3 protects cardiomyocytes from ERS-induced cell death by activating SIRT1 [97].

Hepatocytes synthesize and secrete ceruloplasmin, which participates in the redox process in the body and has both pro- and antioxidative effects. Ceruloplasmin is the main source of serum ferroxidase I. Ferroxidase I is a copper-dependent oxidase that reduces free radicals and catalyzes the conversion of Fe^2+^ to the less toxic Fe^3+^. The post-translational modification of specific proteins, including tyrosine nitrification, is one of the mechanisms through which ROS promotes disease pathogenesis. Studies have reported that nitrified protein and nitro ceruloplasmin levels were upregulated, while ferrous oxidase I was decreased in the total circulation of patients with heart failure. Furthermore, in an isolated cardiac model, it was observed that ceruloplasmin protects against myocardial I/R injury by providing antioxidant activity. Therefore, the redox effect of ceruloplasmin in myocardial tissue requires further investigation [98].

The expression of microRNA (miRNA) is also closely related to the production of ROS, and intracellular ROS can induce or inhibit the expression level of miRNA. The most abundant miRNA in the heart is miR-1, and the appropriate expression of miR-1 is necessary to maintain normal cardiac function. miR-208 is a highly conserved miRNA family, and the overexpression or deletion of miR-208 can cause myocardial hypertrophy, myocardial damage, and arrhythmia. Studies have reported that the activities of antioxidant enzymes (CAT, SOD, and GSH-Px) in patients with heart failure were significantly decreased; the activities of MDA and xanthine oxidase (XOX) were significantly increased; and the inflammatory factors IL-10, iNos, iNOS/eNOS, and TNF-α were significantly increased. In addition, the expression of miR-208 was significantly increased, while the expression of miR-1 was significantly decreased [99]. This suggests that oxidative stress can promote the development of heart failure by changing the expression level of miRNA. Studies have shown that the activity of IRE1α is regulated by the endoplasmic reticulum oxidoreductase PDIA6 (protein disulfide isomerase A6) and miR-322, that is, depletion of Ca^2+^ in the endoplasmic reticulum reduces the abundance of miR-322 and increases the stability of PDIA6 mRNA, thereby increasing the activity of IRE1α in the ERS reaction [100]. Other miRNAs regulate ERS processes, such as increased miR-30b-5p and miR-30c-5p expression and the targeted inhibition of eIF2α protein synthesis, thereby inhibiting the p-eIF2α/ATF4/CHOP proapoptotic pathway and reducing endoplasmic reticulum stress-induced apoptosis. The upregulation of miR23a~27a~24-2 clusters in HEK293T cells leads to increased levels of ATF4 and CHOP and subsequent cell death [101]. These results demonstrate the critical role of miRNAs as key regulators of the endoplasmic reticulum stress response.

Seipin is an intrinsic ER protein with two transmembrane structures, and mutations or deletions of seipin may cause ERS. Moreover, seipin regulates intracellular calcium distribution through the muscle/ER Ca^2+^ pump (Ca^2+^-ATPase, SERCA) in adipocytes [102]. SERCA2a is responsible for the translocation of Ca^2+^ from the cytoplasm to the ER during diastole. In a series of experimental observations in mice following a transverse aortic contraction of seipin gene knockout (SKO), we observed that the expression levels of ERS-related protein GRP78 and inflammatory factors were increased in SKO mice, and Ca^2+^-related factors SERCA2a and P-RYR protein levels decreased [103]. It was indicated that seipin deletion caused diastolic and contractile dysfunction of cardiomyocytes, cardiomyocyte apoptosis, myocardial hypertrophy, and heart failure by activating ERS and Ca^2+^ homeostasis. Apelin (APJ endogenous ligand) is an endogenous ligand of angiotensin receptor-like 1 (APJ). In the pentobarbital sodium-induced rabbit acute heart failure model, apelin decreased GRP78, CHOP, cleaved-caspase-12, cleaved-caspase-3, and B cell lymphoma (Bcl-2)-associated X (Bax) protein levels and improved the Bcl-2 protein level [104]. However, two common ERS agonists, TM and dithiothreitol, blocked the improvement effect of apelin on acute heart failure [104,105].

### 4.3. I/R Injury

There is increasing evidence that ischemia–reperfusion (I/R) injury activates the UPR in cardiomyocytes. During tissue ischemia, the expression of certain ERS response genes (such as GRP78) is induced, owing to a lack of nutrients and oxygen [106], activating UPR progress. GRP78 protects cardiomyocytes from apoptosis induced by I/R injury [107]. Studies have demonstrated that ATF6 activation increased the expression levels of many genes, including GRP78 in transgenic mice expressing activated forms of ATF6 in cardiomyocytes. GRP78 is involved in correctly folding the improperly folded proteins in the ER and reducing myocardial I/R damage [108]. Its ability to reduce myocardial I/R damage stems from the fact that ATF6 is a key transcriptional inducible factor for oxidative stress response genes. The proteins encoded by these genes reduce the production of ROS during I/R, thereby protecting cardiomyocytes and the heart from oxidative stress damage.

The protective effect of GRP78 on I/R tissue is mediated by the nuclear factor-E2-related factor 2 (Nrf2)/HO-1 signaling pathway [107]. Nrf2 and the UPR have protective effects against oxidative stress and ERS. In addition, Nrf2 enters the nucleus to regulate oxidative stress-induced cardiomyocyte apoptosis [109]. The expressions of ERS proteins such as GRP78, PERK, ATF6, XBP1, and CHOP were significantly increased [110]. This indicates that the UPR is an important mechanism of pathological injury after myocardial infarction. By limiting ERS from excessively activating the UPR, cardiomyocyte apoptosis can be reduced and ventricular remodeling and cardiac function can be improved.

### 4.4. Hypertension

In hypertensive diseases, the afterload pressure increases, thus causing heart hypertrophy. The expression of UPR markers GRP78 and GRP94 in the left ventricular myocardial tissue of mildly hypertensive pigs was increased, while the expression level in moderate hypertension was the same as that in normal tissues and even lower than the expression level in mildly hypertensive tissues. This indicates that ERS only briefly manifested in the early stage of left ventricular hypertrophy [111]. UPR participates in the process of cardiac remodeling at a later stage by removing damaged cardiomyocytes via autophagy after using its ER to provide autophagosomes to form the required membranes.

A key regulator of several cellular activity pathways is Rac1. In the vascular system, Rac overexpression alters the redox equilibrium state of the vascular wall and produces ROS by activating Nox [112]. Superoxide and ROS are important mediators in the pathophysiological process of hypertension because they antagonize nitric oxide, which in turn causes superoxide to increase vascular tension [113]. In cardiomyocytes, Rac1 induces cardiomyocyte hypertrophy and regulates the proliferation and migration of vascular smooth muscle cells and the rearrangement of ECs [114]. Blocking Rac1 activation and membrane translocation prevents Nox activation, reduces ROS production, and protects the vascular endothelium [115]. According to the relevant literature, we know that Rac1 is involved in the oxidative stress process, and membrane translocation occurs during Rac1 activation, resulting in increased production of ROS and thus damage to the vascular endothelium. However, there are few studies on the correlation between Rac1 and ERS.

Oxidative stress and ERS/UPR caused by hypoxia are associated with pulmonary hypertension, preeclampsia, and intrauterine growth retardation [116]. Cell dysfunction and apoptosis result from the persistent activation of hypoxia-inducible factors, ROS, and UPR. In the chronic hypoxic mouse pulmonary hypertension model, all UPR pathways are activated, and the expressions of ATF6, PERK, IRE1α, and CHOP increase, indicating that ERS is involved in the occurrence and development of pulmonary hypertension [117].

### 4.5. Cardiomyopathy

DCM is the most common disease of diabetes. It is a chronic and complex process, and the relationship between DCM and ERS has often attracted our attention. Multiple studies have demonstrated that hyperglycemia can cause mitochondrial dysfunction in cardiac tissue and ERS, which promotes the activation of apoptotic pathways and may be connected to the production of ROS, which is mainly produced by mitochondria and Nox in cardiomyocytes [118,119,120,121]. In addition, hyperglycemia induces advanced glycation end products (AGEs) and inflammatory responses. AGEs and ROS initiate pro-inflammatory responses by activating NF-κB in B cells [122].

MT is a potent, non-specific antioxidant that clears a variety of ROS and/or reactive nitrogen species. In MT-TG diabetic mice, there was no increase in cardiac ERS or apoptosis; however, there were significant increases in cardiac ERS-related proteins, such as GRP78, GRP94, cleaved ATF6, p-eIF2α, CHOP, caspase-3, and caspase-12, which in turn increased apoptosis [118,123]. This demonstrates that diabetes can cause cardiac ERS, while MT can prevent it. In addition, studies have shown that the ubiquity of MT in the body can prevent diabetes-induced ERS and MT synthesis, and diabetes-induced ERS can increase cardiac MT synthesis [123]. Under the condition of high glucose, H9C2 cells were treated with granulocyte colony-stimulating factor (G-CSF), and it was observed that G-CSF significantly downregulated GRP78, caspase-9, caspase-12, IRE-1α, and CHOP, thereby reducing the apoptosis of cardiomyocytes and neutrophils in DCM [124]. LCZ696, synthesized from valsartan and sacubitril, inhibits the overactivation of the renin–angiotensin–aldosterone system while increasing the natriuretic peptide system for cardiovascular protection. LCZ696 alleviates DCM by inhibiting myocardial inflammation, ERS, and apoptosis through the AGEs/NF-κB and PERK/CHOP cascade signaling pathways. ERS was observed in diabetic mice. The mRNA and protein levels of GRP78, PERK, eIF2a, ATF4, and CHOP were downregulated, while LCZ696 could reverse this trend [122]. Other antioxidant extracts, such as astragalus polysaccharides, rutin, and tanshinone IIA, inhibited the expression levels of p-PERK, ATF6, and CHOP [125,126,127]. Exercise training improved DCM in diabetic rats by significantly decreasing GRP78, CHOP, and cleaved caspase-12 protein expression and inhibiting ERS-induced apoptosis [128].

**Figure 4:**
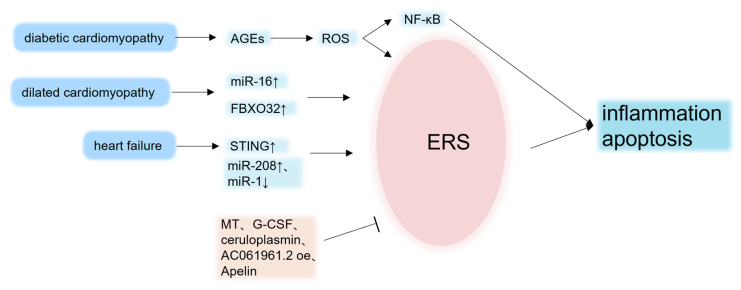
Endoplasmic reticulum stress, heart failure, and cardiomyopathy. In heart failure and cardiomyopathy, ERS is caused by different mechanisms, causing inflammation and apoptosis, which aggravate the lesion. Some metal-binding proteins or different drugs inhibit the ERS process and improve cardiac function. AGEs, advanced glycation end products; FBXO32, F-Box Protein 32; STING, stimulator of interferon genes; MT, sincemallothionein; G-CSF, granulocyte colony-stimulating factor; NF-κB, nuclear factor kappa-B; arrow, promote; flat arrow, suppress. The up arrows indicate increase and the down arrows indicate decrease.

The apoptosis of cardiomyocytes causes a decrease in viable cardiomyocytes and the hypertrophy of pathological cardiomyocytes, which can lead to changes in cardiac cavity structure and ventricular remodeling, ultimately leading to dilated cardiomyopathy. In dilated cardiomyopathy model rats, β-catenin, Axin2, c-Myc, and Bcl-2 levels decreased, whereas GRP78, CHOP, caspase-3, and Bax levels increased, and ERS-induced cardiomyocyte apoptosis increased. However, the overexpression of AC061961.2 can inhibit ERS-induced cardiac dysfunction and cardiomyocyte apoptosis in dilated cardiomyopathy rats by activating the Wnt/β-catenin pathway [129]. miRNAs play a key role in the development and repair of the cardiovascular system. The overexpression of miR-16 promotes UPR activation by increasing PERK, ATF4, and CHOP mRNA expression and accelerating ERAD responses (by increasing EDEM and OS-9 gene expression), which in turn induce apoptosis and decrease angiogenesis. However, the protein and mRNA levels of GRP78, ATF6, and XBP1 are downregulated. The authors believe that this downregulation trend may be owing to ATF6 being a direct target of miR-16 or the inhibition of protein translation by the PERK/CHOP pathway [130]. FBXO32 is a muscle-specific ubiquitin-E3 ligase. A major imbalance in protein ubiquitination caused by FBXO32 mutations results in the early onset of human cardiomyopathy. CHOP mediates the expression of pro- and anti-apoptotic proteins, such as Bcl-2, Bcl-XL, BIM, and GADD3425, causing a significant increase in apoptosis. In addition, abnormally increased levels of ATF2 are produced [131], which ultimately promotes the development of dilated cardiomyopathy (Figure 4).

## 5. Conclusions

The normal biological function of cells depends on the biological function of proteins, which in turn are inseparable from the ER, the membrane organelle in the cell. The ER is responsible for processing, secreting, and transporting proteins to the Golgi body, lysosome, or plasma membrane to perform their respective functions under normal physiological conditions. However, under certain stress conditions, an excessive number of misfolded proteins accumulate in the ER, causing ERS, which in turn causes disturbances in the level or function of related proteins, ultimately leading to a series of diseases. In conclusion, we understand that calcium homeostasis imbalance and ERS regulate each other, and ERS can induce the activation of embryonic genes in adult cardiomyocytes and thus serve a protective role on cardiomyocytes. In the cardiovascular system, myocardial injury increases the expression of ERS-related proteins, which target the degradation of misfolded proteins in an attempt to repair ER function. When UPR is sustained over time due to damaging factors, a series of signaling pathways eventually induce apoptosis. The expression of the marker CHOP increases apoptosis and accelerates the degree of myocardial damage. Therefore, it is essentially crucial to investigate the mechanism of ERS and its relationship with diseases. Theoretically, we understand that metal-binding proteins play an important role in inhibiting ERS. For example, selenoprotein, zinc transporters, ferritin, ceruloplasmin, and MT have antioxidant effects. However, more studies on cardiovascular diseases are required to confirm that metal-binding proteins can protect the heart by inhibiting ERS.

## Figures and Tables

**Table 1:** Trace elements and cardiovascular pathological changes.

Reference	Trace Element	Cardiovascular Changes	Mechanism Mediated by Trace Element/Metal-Binding Proteins
Liu et al. [30]	selenium	atherosclerosis	Selenium inhibits endoplasmic reticulum stress and activation of PI3K/AKT and ERK signaling pathways, regulates inflammation, and then inhibits vascular calcification and apoptosis
Liu et al. [31]			
Lu et al. [32]			SelK overexpression attenuates intracellular reactive oxygen species levels and protects cells from oxidative stress-induced toxicity in cardiomyocytes
Meiler et al. [33]			SelK enhances the expression and distribution of CD36 on the plasma membrane. SelK deficiency can reduce LDL uptake and foam cell formation, promote foam cell formation, and promote the occurrence of atherosclerosis
Lewis et al. [34]			Macrophages, α-smooth muscle actin, RAGE receptors, VCAM-1, and CTGF are increased and atherosclerotic lesions are increased in diabetic mice lacking GPx1 and ApoE
Feng et al. [35]		heavy metal toxicity (cadmium)	Selenium inhibits oxidative stress and programmed cell death via PI3K/AKT/PTEN signaling pathway
Gao et al. [36]		heavy metal toxicity (mercury)	Selenium attenuates HgCl_2_-induced ERS and apoptosis, restores Ca^2+^ homeostasis, and attenuates oxidative stress in cardiomyocytes
Wang et al. [37]		cardiomyopathy	Selenium deficiency causes Ca^2+^ homeostasis imbalance in the endoplasmic reticulum, significantly reduces GPx activity, increases ROS production, triggers endoplasmic reticulum stress, and increases CHOP and GRP18
Al-Mubarak et al. [38]		ischemia and reperfusion	Inhibits oxidative stress and apoptosis, senses luminal calcium levels, and thereby regulates SERCA-mediated replenishment of ER calcium stores
Chen et al. [39]		Keshan disease	Selenium deficiency reduces cytoprotective autophagy in primary cardiomyocytes
Xu et al. [40]			Selenium deficiency reduces myocardial Gpx1 activity, causes ERS, and promotes GRP78, CHOP, and p-eIF2α expression
Sun et al. [41]		heart failure	Selenium inhibits oxidative stress-induced cell cycle arrest by activating the PI3K/AKT signaling pathway
Zhao et al. [42]	zinc	ischemia and reperfusion	Inhibition of the “endoplasmic reticulum stress/CaMKII/STAT3 pathway” to protect myocardium
Dabravolski et al. [43]			Zinc protects the heart from H/R injury by preventing mitochondrial superoxide production and dissipation of mitochondrial membrane potential by inducing autophagy and mitochondrial autophagy by increasing ERK activity and Beclin1 expression and stabilizing PINK1
Rosenblum et al. [44]		heart failure	Zinc deficiency causes oxidative stress, cardiac hypertrophy, and significant degeneration and massive fibrosis of cardiomyocytes
Wang et al. [45]		myocardial hypertrophy	Zinc deficiency leads to activation of BCL10/CARD9/p38MAPK signaling pathway and induces obstruction-related cardiac hypertrophy
Olgar et al. [46]			Significantly increased expression of ZIP7, ZIP14, and ZnT8, and decreased levels of ZIP8 and ZnT7 in myocardial hypertrophy
Degirmenci et al. [47]		arrhythmia	High-[Zn^2+^]_i_ induces significant activation of ATP-sensitive K^+^-channel currents and zinc plays an important role in cardioprotection through changes in cellular ATP and sulfur oxygenation levels
Bodiga et al. [48]		hypoxia–reoxygenation	Zinc supplementation inhibits NOX2 mRNA, reduces oxidative stress and endoplasmic reticulum stress, and reduces cell death
Lu et al. [49]		diabetic cardiomyopathy	Zinc supplementation inhibits autophagy and endoplasmic reticulum stress: myocardial content of LC3 and GRP78 proteins is significantly reduced
Wang et al. [50]		ischemia/reperfusion	Inhibition of mPTP opening, inhibition of ERS, GRP 78 and GRP 94 expression decline, and protect the heart
Song et al. [51]		diabetic cardiomyopathy	Involvement of SOD and thioredoxin in enzyme activity and chelator activity, stabilization of cell membranes, and inhibition of lipid peroxidation thioredoxin
Cai et al. [52]	metallothionein	diabetic cardiomyopathy	MT binds Zn^2+^ under physiological conditions and releases Zn^2+^ under diabetes-induced oxidative stress
Cai et al. [53]			MT inhibits mitochondrial GSH depletion, cardiac protein nitration, lipid peroxidation, and cardiac cell apoptosis
Zhou et al. [54]		aortic lesions caused by IH	MT inhibits oxidative damage, inflammatory response, and apoptosis

SelK, selenomins K; ApoE, apolipoprotein E; GPx, glutathione peroxidase; RAGE, advanced glycation end products; VCAM-1, vascular cell adhesion molecule-1; VEGF, vascular endothelial growth factor; CTGF, connective tissue growth factor; MT, metallothionein; IH, intermittent hypoxia; GSH, glutathione.

## Data Availability

Not applicable.

## Abbreviations

The following abbreviations are used in this manuscript:

Abbreviation	Meaning
FAs	fatty acids
ER	endoplasmic reticulum
ROS	reactive oxygen species
ERS	endoplasmic reticulum stress
MT	metallothionein
UPR	unfolded protein response
PERK	protein kinase RNA-like ER kinase
eIF2a	eukaryotic initiation factor 2α
ATF4	activating transcription factor 4
ATF6	activating transcription factor 6
IRE1	inositol-requiring enzyme type 1
XBP1	X-box-binding protein 1
GRP78	78 kDa glucose-regulated protein
SOD	superoxide dismutase
CAT	catalase
GSH-Px	glutathione peroxidase
Sep15	selenomins 15
SelS	selenomins S
SelN	selenomins N
SelK	selenomins K
SelT	selenomins T
ERAD	ER-associated protein degradation
Sepp1	selenoprotein P
Txnrd2	thioredoxin reductase 2
ATP	adenosine triphosphate
DCM	diabetic cardiomyopathy
I/R	ischemia-reperfusion
MDA	malondialdehyde
TPEN	Zn^2+^ chelators *N*,*N*,*N*′,*N*′-tetrakis (2-pyridylmethyl)-ethylenediamine
Nox2	NADPH oxidase 2
mPTP	mitochondrial permeability transition pore
TM	tunicamycin
GSK-3β	glycogen synthase kinase-3β
ERK	extracellular signal-regulated kinase
Mn	manganese
ID	iron deficiency
ECs	endothelial cells
ICAM	intercellular adhesion molecule
VCAM	vascular cell adhesion molecule
TNFα	tumor necrosis factor α
IFN-γ	interferon-γ
STIM1	stromal interaction molecule 1
STIM2	stromal interaction molecule 2
SOCE	store-operated calcium entry
ORAI	calcium channel protein
LDL	low-density lipoprotein
NLRP3	nucleotide-binding receptor protein 3
NOD	nucleotide-binding domain
STING	stimulator of IFN genes
SIRT1	silent information regulator 1
SKO	seipin gene knockout
APJ	angiotensin receptor-like 1
Apelin	APJ endogenous ligand
AGEs	advanced glycation end products
G-CSF	granulocyte colony-stimulating factor
